# Ginger Is a Potential Therapeutic for Chronic Toxoplasmosis

**DOI:** 10.3390/pathogens11070798

**Published:** 2022-07-15

**Authors:** Asmaa M. El-kady, Wafa Abdullah I. Al-Megrin, Iman A. M. Abdel-Rahman, Eman Sayed, Eman Abdullah Alshehri, Majed H. Wakid, Fadi M. Baakdah, Khalil Mohamed, Hayam Elshazly, Hussah M. Alobaid, Safa H. Qahl, Hatem A. Elshabrawy, Salwa S. Younis

**Affiliations:** 1 Department of Medical Parasitology, Faculty of Medicine, South Valley University, Qena 83523, Egypt; 2 Department of Biology, College of Science, Princess Nourah bint Abdulrahman University, P.O. Box 84428, Riyadh 11671, Saudi Arabia; waalmegrin@pnu.edu.sa; 3 Department of Pharmacognosy, Faculty of Pharmacy, South Valley University, Qena 83523, Egypt; emanabdelraheem@svu.edu.eg; 4 Department of Parasitology, Faculty of Veterinary Medicine, South Valley University, Qena 83523, Egypt; emy.hosam@yahoo.com; 5 Department of Zoology, College of Science, King Saud University, Riyadh 11362, Saudi Arabia; ealshehri@ksu.edu.sa (E.A.A.); hesalobaid@ksu.edu.sa (H.M.A.); 6 Department of Medical Laboratory Technology, Faculty of Applied Medical Sciences, King Abdulaziz University, Jeddah 21589, Saudi Arabia; mwakid@kau.edu.sa (M.H.W.); fbaakdah@kau.edu.sa (F.M.B.); 7 Special Infectious Agents Unit, King Fahd Medical Research Center, King Abdulaziz University, Jeddah 21589, Saudi Arabia; 8 Department of Epidemiology, Faculty of Public Health and Health Informatics, Umm Al-Qura University, Mecca 21961, Saudi Arabia; kmismail@uqu.edu.sa; 9 Department of Biology, Faculty of Sciences -Scientific Departments, Qassim University, Buraidah, Qassim 52571, Saudi Arabia; hayam.elshazly@yahoo.com; 10 Department of Zoology, Faculty of Science, Beni Suef University, Beni Suef 62521, Egypt; 11 Department of Biology, College of Science, University of Jeddah, Jeddah 21959, Saudi Arabia; shqahal@uj.edu.sa; 12 Department of Molecular and Cellular Biology, College of Osteopathic Medicine, Sam Houston State University, Conroe, TX 77304, USA; 13 Department of Medical Parasitology, Faculty of Medicine, Alexandria University, Alexandria 21131, Egypt; younis_salwa@yahoo.com

**Keywords:** toxoplasmosis, *T. gondii*, ginger, cysts, brain, lung, liver, treatment

## Abstract

**Background:***Toxoplasma gondii* (*T. gondii*) is an opportunistic parasite that causes serious diseases in humans, particularly immunocompromised individuals and pregnant women. To date, there are limited numbers of therapeutics for chronic toxoplasmosis which necessitate the discovery of effective and safe therapeutics. In the present study, we aimed to evaluate the antitoxoplasmosis potential of ginger extract in mice with experimentally induced chronic toxoplasmosis. **Results:** Treatment with ginger extract significantly reduced cysts count in the brains of *T. gondii*-infected mice with a marked alleviation of edema and inflammation, and a reversal of neuronal injury. Moreover, ginger extract treatment reduced inflammation in liver and lungs and protected hepatocytes from infection-induced degeneration. Consistently, apoptosis was significantly mitigated in the brains of ginger extract-treated mice compared to infected untreated animals or spiramycin-treated animals. **Methods:** Four groups of Swiss albino mice (10 mice each) were used. The first group was not infected, whereas 3 groups were infected with Me49 *T. gondii* strains. One infected group remained untreated (infected untreated), whereas the other two infected groups were treated with either ginger extract (250 mg/kg) or spiramycin (positive control; 100 mg/kg), respectively. The therapeutic potential of ginger extract was evaluated by calculation of the parasite burden in infected animals, and examination of the infected tissues for reduced pathologic changes. **Conclusions:** Our results showed for the first time that ginger extract exhibited marked therapeutic effects in mice with chronic *T. gondii* infection which indicates that it can be used as a safe and effective treatment for chronic toxoplasmosis.

## 1. Introduction

*Toxoplasma gondii* (*T. gondii*) is an obligate intracellular protozoan parasite which can infect nearly all warm-blooded animals, including humans [1,2,3,4]. Current estimates indicate that around one-third of the world’s population has a serologically positive toxoplasmosis test [5].

Humans are primarily infected with *T. gondii* following ingestion of undercooked meat containing viable tissue cysts, and water or food contaminated with oocysts in infected cat feces [6,7,8,9,10]. Infection with *T. gondii* is usually asymptomatic in healthy individuals; however, it could lead to serious disease in immunocompromised individuals, such as HIV/AIDS patients, cancer patients, and organ transplant recipients, and in pregnant women with the possibility of its being transmitted to the fetus [11,12,13,14].

Currently, there are limited effective therapeutic options for toxoplasmosis with no optimal effective treatment for chronic toxoplasmosis due to poor penetration into the brain and potential side effects [15,16,17,18,19]. The combination of pyrimethamine and sulfadiazine (pyr-sulf), targeting the active stage of the infection and suppressing parasite multiplication in the early stages of the disease, is the current gold standard for treating toxoplasmosis [15,16]. However, several side effects have been reported for this medication including neutropaenia, decreased platelet count, thrombocytopaenia, leucopaenia, elevated serum creatinine and liver enzymes, and hypersensitivity reactions [20]. Although other treatment regimens have been used including pyrimethamine in combination with clindamycin, atovaquone, clarithromycin, or azithromycin, none has been found to be superior to pyr-sulf or active against the latent stage of the infection [16].

Based on the limited treatment options and potential side effects, several research groups have considered the use of medicinal plant extracts as potential alternative and safe therapeutics for toxoplasmosis [21]. Active constituents from medicinal plants have demonstrated diverse pharmacological activities including antiprotozoal, antibacterial, anti-inflammatory, and other activities including immunomodulatory activities [22,23]. *Zingiber officinale*, commonly known as ginger, is a familiar dietary spice with multiple reported pharmacological activities. It has long been widely used as a common household remedy, flavoring agent, antiemetic, and as a digestive aid [24]. Ginger contains several active ingredients including volatile oil, gingerols, shogaols, paradols, gingerdiols, and zingerone which are responsible for the anti-inflammatory, antidiarrheal, antibacterial, antiviral, antifungal, and antioxidant properties [25]. In addition to the aforementioned activities, several studies have demonstrated the antiparasitic activities of ginger against *Schistosoma* spp., *Trichinella spiralis*, microfilaria of *Dirofilaria immitis* and protoscolices of hydatid cyst [26,27,28,29]. Recently, new anti-*T. gondii* medications were evaluated mainly for the treatment of acute toxoplasmosis induced by the RH strain but not for treatment of chronic toxoplasmosis [30].

In the present study, we evaluated the potential therapeutic activity of ginger extract against chronic *T. gondii* infection induced by the Me49 strain in experimentally infected mice. Our data showed that ginger extract is an effective and potential therapeutic option for chronic toxoplasmosis.

## 2. Results

### 2.1. Treatment with Ginger Extract Significantly Reduced Cyst Count in the Brains of T. gondii-Infected Mice

**Figure 1:**
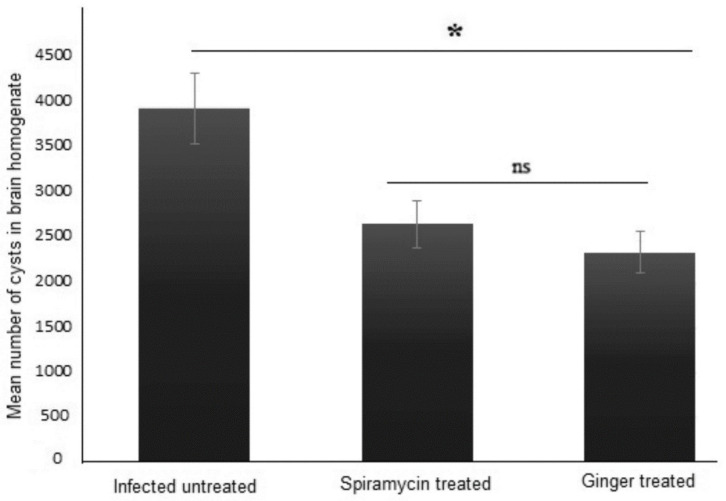
**Ginger extract treatment significantly reduced cysts count in brains of infected mice.** Total number of cysts was counted in brain homogenates of infected untreated, sprimaycin-treated, and ginger extract-treated mice (5 mice/group). Data are expressed as means with error bars representing SD and were analyzed using ANOVA. Asterisk (*) indicates a significant difference in the numbers of cysts in treated groups compared to the infected untreated group (*p* = 0.001), and “ns” indicate insignificant difference.

The therapeutic effect of ginger extract against chronic toxoplasmosis was evaluated by measuring the *T. gondii* cyst burden in the brains of infected mice. Treatment with ginger extract or spiramycin (positive control) significantly reduced the number of *T. gondii* cysts in brains of mice with chronic toxoplasmosis compared to infected untreated animals (*p* = 0.001; Figure 1).

### 2.2. Treatment with Ginger Extract Protected Brain and Reduced Infection-Induced Edema and Inflammation

**Figure 2:**
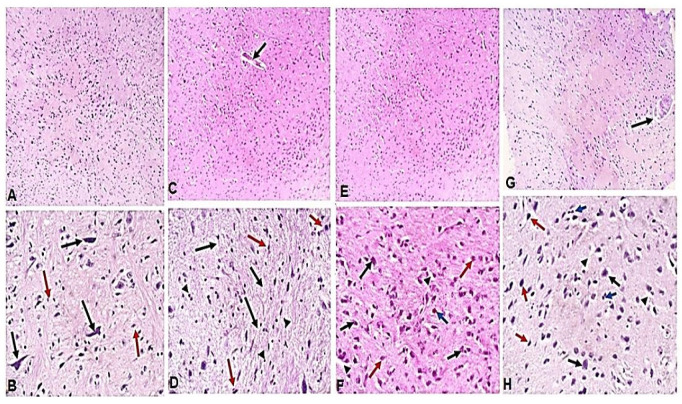
**Treatment with ginger extract reversed pathological changes in brains of *T. gondii*-infected mice.** Sections of mice brains of different groups were stained with H & E and imaged at 100× and 400× magnification. (**A**) Representative image (100×) of brain tissue section of uninfected mice showing uniform brain tissue. (**B**) Higher magnification (400×) of (**A**) showing uniform normal neurons (black arrows) and glial tissue (red arrows). (**C**) Representative image (100×) of brain tissue section of infected untreated mice with clear *T. gondii* cyst (black arrow). (**D**) Higher magnification (400×) of (**C**) showing brain edema (black arrows) and chronic inflammatory cell infiltrate (arrowheads), and red neurons (red arrows). (**E**) Representative image (100×) of brain sections of spiramycin-treated mice. (**F**) Higher magnification (400×) of (**E**) illustrating uniform neurons (black arrows), mild edema (arrowheads), proliferating glial cells (red arrows), and a red neuron (blue arrow). (**G**) Representative image (100×) of brain tissue sections of infected ginger extract-treated mice showing degenerated cyst (black arrow). (**H**) Higher magnification (400×) of (**G**) clearly demonstrating uniform neurons (black arrows), absence of red neurons, significantly reduced edema (arrowheads), reduced inflammatory cellular infiltration (blue arrows), and marked proliferation of glial cells (red arrows).

Next, H&E-stained brain sections were used to examine the therapeutic effect of ginger extract in the alleviation of pathological changes in the brains of mice with chronic toxoplasmosis. Brain tissues of uninfected mice showed uniform normal neurons (black arrows) within glial tissue (red arrows) (Figure 2A,B). On the other hand, brain tissues of infected untreated animals showed *T. gondii* cyst (Figure 2C, black arrow) with marked brain edema (Figure 2D, black arrows) and chronic inflammatory cell infiltrate (Figure 2D, arrowheads). Prominent red neurons were observed (Figure 2D, red arrows) which indicate acute neuronal injury and subsequent apoptosis or necrosis but no glial proliferation or gliosis. On the other hand, brain sections of spiramycin-treated mice showed uniform neurons (black arrows) with mild edema (arrowheads), proliferated glial cells and gliosis (red arrows), and a single red neuron (blue arrow), which indicate protection of the brain against necrosis or apoptosis (Figure 2F). Interestingly, ginger extract-treated mice showed significant restoration of the brain architecture with uniform neurons (black arrows) and a complete absence of red neurons. Moreover, a significant alleviation of edema (arrowheads) and inflammatory cellular infiltration (blue arrows) was observed with a marked proliferation of glial cells (red arrows) (Figure 2H).

### 2.3. Treatment with Ginger Extract Preserved the Liver and Reduced Inflammation in T. gondii-Infected Mice

**Figure 3:**
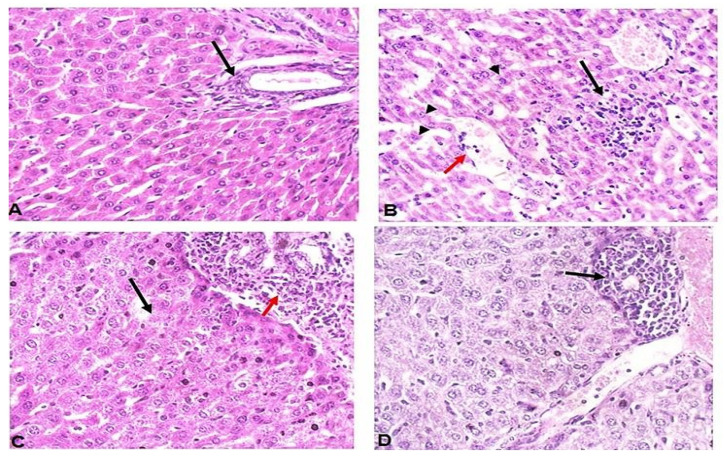
**Ginger extract treatment protected the liver of *T. gondii*-infected mice against infection- induced pathologies.** (**A**) Representative image of liver tissue sections of uninfected mice stained with H&E showing normal hepatocytes and portal tract (black arrow). (**B**) Representative image of liver tissue sections of infected untreated mice with clear lobular inflammation (black arrow), vascular congestion (red arrows) and degeneration of hepatocytes (arrow heads). (**C**) Representative image of liver tissue sections of spiramycin-treated mice showing lytic necrosis (black arrow), inflammation (red arrow), and hydropic hepatocytes degeneration. (**D**) Representative image of liver tissue sections of ginger extract-treatment showing uniform preserved hepatocytes, mild lobular inflammation (black arrow), and no vascular congestion. All images were taken at 400× magnification.

Unlike uninfected mice which showed uniform hepatocytes and a portal tract (black arrow) (Figure 3A), liver tissues of infected untreated mice (Figure 3B) showed lobular inflammation (black arrow), vascular congestion (red arrows) and hydropic degeneration of hepatocytes (arrow heads). On the other hand, liver tissues of spiramycin-treated mice showed lytic necrosis (black arrow), portal tract expansion of chronic inflammatory cells (red arrow), and hydropic degeneration of hepatocytes (Figure 3C). Interestingly, ginger extract-treatment preserved the liver, which showed uniform hepatocytes and mild lobular inflammation (black arrow) with no vascular congestion (Figure 3D).

### 2.4. Treatment with Ginger Extract Reduced Inflammation in Lungs of T. gondii-Infected Mice

**Figure 4:**
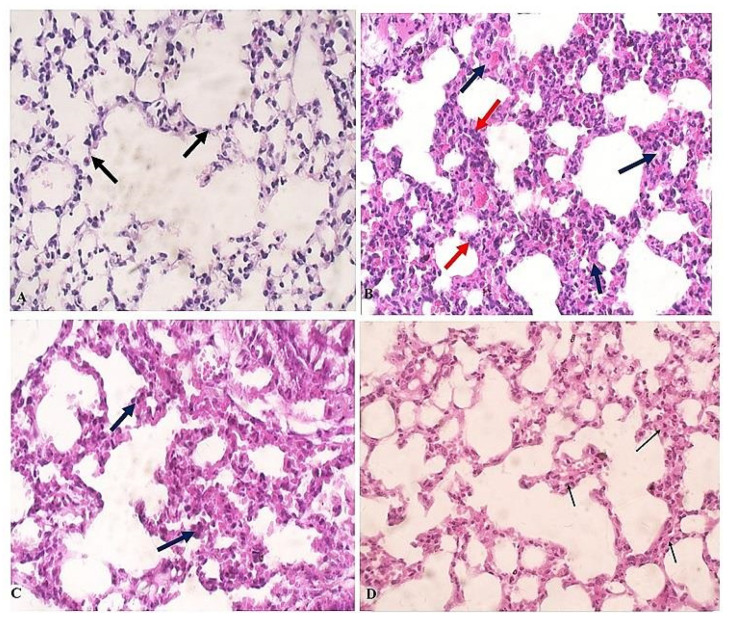
**Ginger-extract-treatment mitigated inflammation and reduced thickness of alveolar septa in lungs of *T. gondii*-infected mice**. (**A**) Representative image of lung tissue sections of uninfected mice stained with H&E showing uniform alveolar tissue with normal alveolar septa (black arrows). (**B**) Representative image of lung tissue sections of *T. gondii*-infected untreated mice showing significant thickening of alveolar septa with significant inflammatory infiltrate (red arrows) and edema (black arrows). (**C**) Representative image of lung tissue sections of infected mice treated with spiramycin showing thickened alveolar septa and chronic inflammatory cells (black arrows). (**D**) Representative image of lung tissue sections of infected mice treated with ginger extract showing alveolar septa with reduced thickening and inflammation (black arrows). All images were taken at 400× magnification.

Lung tissues of uninfected animals showed no signs of inflammation and normal alveolar septa (Figure 4A, black arrows). In contrast, infected untreated animals showed significantly thickened alveolar septa with inflammatory infiltrate (Figure 4B, red arrows) and edema (Figure 4B, black arrows). However, treatment with spiramycin did not significantly reduce the inflammatory infiltrate and the thickening of the alveolar septa (Figure 4C, black arrows). However, ginger extract treatment markedly reduced the inflammation and the thickening of the alveolar septa (Figure 4D, black arrows).

### 2.5. Treatment with Ginger Extract Protected Brains of T. gondii-Infected Mice from Apoptosis

**Figure 5:**
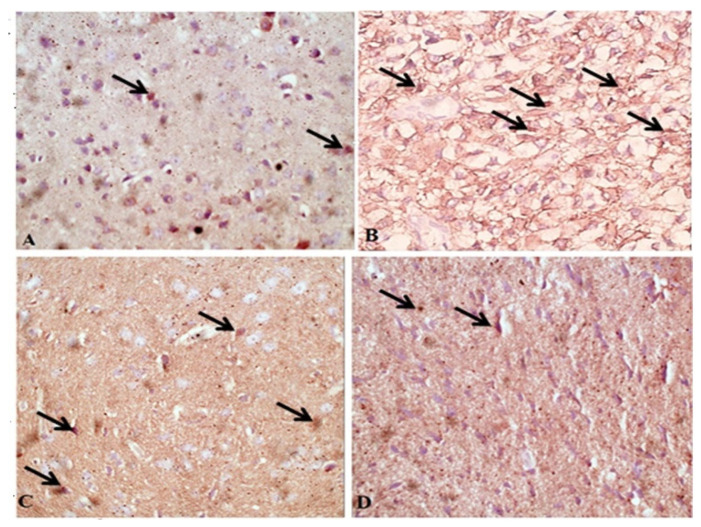
**Treatment with ginger extract reduced caspase-3 levels in brain cells of *T. gondii*-infected mice.** IHC representative images of brain tissue sections stained for caspase-3 showing low levels in uninfected mice (**A**), and higher levels in infected untreated mice (**B**). Treatment with spiramycin (**C**) or ginger extract (**D**) reduced neuronal caspase-3 levels compared to infected untreated mice. All images were taken at 400× magnification.

Next, the protective effect of ginger treatment against infection-induced apoptosis of brain cells was examined using caspase-3 staining. Immunohistochemical staining of brain tissue sections demonstrated very weak caspase-3 staining in neurons of uninfected mice (Figure 5A). In contrast, cerebral cortical neurons of infected untreated mice showed significantly higher intensity of caspase-3 compared to uninfected animals (Figure 5B; *p* = 0.003). However, treatment with either spiramycin (Figure 5C) or ginger extract (Figure 5D) significantly reduced neuronal caspase-3 levels compared to infected untreated mice, which indicates the protective effect of treatments against apoptosis.

**Figure 6:**
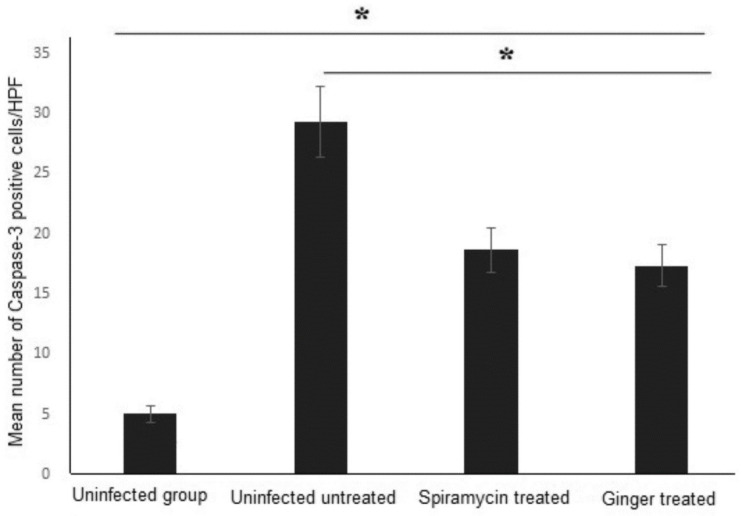
**Treatment with ginger extract significantly reduced the number of caspase-3 positive cells in brains of *T. gondii*-infected mice**. The mean number of caspase-3-positive cells/HPF in the brain tissue sections of each group of mice was calculated and compared. Asterisks (*) indicate a significant difference; *p* < 0.05.

Moreover, significantly higher number of caspase-3 positive cells/HPF were detected in brains of infected untreated mice compared to uninfected mice (Figure 6). Interestingly, ginger extract and spiramycin treatments of infected mice significantly reduced the number of caspase-3 positive cells (Figure 6). These results demonstrate the efficacy of ginger extract in protecting the brain against *T. gondii*-induced apoptosis of brain cells.

## 3. Discussion

*T. gondii* infects about one-third of the world’s population and causes serious complications in immunocompromised patients and pregnant women [31,32]. To date, medications used for the treatment of toxoplasmosis are either ineffective in the chronic stage of the disease or have several side effects [33,34,35]. Furthermore, the majority of novel ani-*T. gondii* drugs were evaluated in the acute phase of infection [36]. Therefore, the discovery of effective and safe medications against the chronic phase of the disease is a necessity. Active constituents in medicinal plants including ginger have demonstrated diverse pharmacological activities such as antibacterial, antiviral, and antifungal activities. However, a few reports have evaluated the antiparasitic activities of ginger, particularly in the treatment of toxoplasmosis. In these studies, ginger extract was only tested for its antiparasitic effect in an acute murine toxoplasmosis murine model induced by infection with *T. gondii* RH strain [14,21,36,37]. In the present study, we evaluated the therapeutic potential of ethanolic extract of ginger in a chronic toxoplasmosis murine model induced by oral infection with an Me49 strain of *T. gondii*.

Our results demonstrated that ginger extract treatment resulted in a significant reduction in the number of *T. gondii* cysts in the brains of infected mice compared with infected untreated animals. Unlike other studies which reported the efficacy of ginger extract only in acute toxoplasmosis [21,37], our study is the first to report the effectiveness of ginger in chronic toxoplasmosis. In another study by Amir et al., silver nanoparticles in a ginger extract base were tested for their in vitro efficacy against tachyzoites of the RH strain of *T. gondii* [14]. In line with Choi et al., they demonstrated a marked lethal effect of the extract on tachyzoites.

In agreement with our results, several studies have demonstrated the antiparasitic efficacy of ginger extract against multiple parasites such as *Schistosoma*, *T. spiralis*, *D. immitis*, *Plasmodium*, *Giardia* and *Trypanosomes* [23,38,39,40,41,42,43].

Our histopathological examination of liver, lung and brain tissues revealed that ginger extract treatment resulted in a marked alleviation of histopathological changes and inflammation induced by *T. gondii* infection. A histopathological examination of the brains of infected untreated mice showed pathological lesions which included *T. gondii* cysts, edema, red neurons and chronic inflammatory cell infiltrate. We believe that these lesions are caused by a disruption of blood–brain barrier and invasion of brain tissue by the parasite [15]. *T. gondii* infection-induced pathological changes in the brain are potentially due to high oxidative stress, high levels of nitric oxide production, glial activation and apoptosis [4,15,44,45,46]. In the present study, treatment of *T. gondii*-infected mice with ginger extract resulted in a marked improvement of *T. gondii* infection-induced brain pathological changes. Brain sections from ginger extract-treated mice showed reduced inflammatory cellular infiltrate, uniform neurons, an absence of red neurons, increased glial cells (gliosis), and reduced apoptosis [15]. 

Glial cells have been thought to play a major role in host defense against *T. gondii* by secreting cytokines such as IL-1, IL-6, GM-CSF, IL-10, IFN-gamma, and chemotactic cytokines [47,48]. We believe that ginger extract can affect the host’s response to the *T. gondii* ME-49 strain in a chronic brain infection, presumably by modifying inflammatory response. This has been supported by Hussein et al., who reported that ginger has neuroprotective effects due to its high content of polyphenolic compounds [49]. Moreover, another study reported that ginger protected the brain of diabetic rats by reducing oxidative stress, inflammation, and apoptosis [50]. The authors also reported that ginger reduced acetylcholinesterase (AchE) expression, modulated the astroglial response to injury, and promoted neurogenesis [50]. Moreover, it is well documented that anti-inflammatory medications that block cyclooxygenase-2, such as ginger, reduce neuropathology in *T. gondii*-infected mice [51,52].

*T. gondii* infections are well known to inhabit organs other than the brain, particularly the liver. To evaluate the therapeutic effect of ginger extract on the liver in chronic toxoplasmosis, we performed a histological examination of liver tissue from every group of mice. Our results showed that hepatic tissues of infected untreated mice demonstrated moderate to severe lobular inflammation (mostly lymphocytes and plasma cells), hydropic degeneration, and congested vessels [53,54,55,56]. Interestingly, ginger extract treatment completely reversed *T. gondii*-induced pathologic changes, restored normal hepatocytes, significantly reduced inflammatory infiltrate, and prevented hydropic degeneration and lytic necrosis. Ginger extract treatment demonstrated higher therapeutic efficacy because liver tissues of spiramycin-treated animals still showed a portal tract expansion with inflammatory cells and a mild degree of hydropic degeneration.

Pulmonary toxoplasmosis has been previously reported in naturally infected animals and in human cases [57,58,59,60]. In the present study, we examined the lung tissue of all animal groups to evaluate the therapeutic effect of ginger against pulmonary toxoplasmosis. We found significant histopathological changes in the form of thickening of the alveolar septa and infiltration of chronic inflammatory cells in infected untreated animals. Hassanein et al. reported similar findings in acute and chronic murine toxoplasmosis [61]. Similar results were also reported in a naturally infected cat [57]. On the other hand, we showed that treatment with ginger resulted in a marked improvement of *T. gondii*-induced pulmonary lesions. Interestingly, the improvement was more significant in ginger- than spiramycin-treated animals. Our results are consistent with other studies which showed that ginger efficiently reduced lung damage and protected lungs from severe damage due to hyperoxia, bronchial asthma, and chronic inflammation [62,63,64].

*T. gondii* has been observed to induce apoptosis of the host cell [65,66]. Significantly high levels of caspase 3, caspase 8, and caspase 9 were recognized in the brain tissues of infected animals, indicating significant apoptosis of brain cells [67]. In the present study, we examined the brain sections of mice to evaluate the potential effect of ginger extract in reducing apoptosis of brain tissue in *T. gondii*-infected animals. Similar to previous studies which documented the ability of ginger to reduce the numbers of apoptotic cells, we demonstrated a significant reduction in the number of caspase-positive cells in ginger extract-treated mice which may be attributed to the efficacy of the extract in reducing the number of tissue cysts. [68,69].

## 4. Materials and Methods

### 4.1. Plant Material and Extract Preparation

The dried rhizome of *Zingiber officinale* (ginger) was purchased from the local market at Qena Governorate, Egypt. Botanical identification was done at the Department of Pharmacognosy, Faculty of Pharmacy, South Valley University, Qena, Egypt. A voucher specimen of the plant (code: Zo.82) was kept in the herbarium, Department of Pharmacognosy, Faculty of Pharmacy, South Valley University, Egypt. The plant was ground to a fine powder using a dry electric mill, then sieved and stored in a sealed dark container until use.

Ginger ethanolic extract was prepared by maceration as previously described [70]. Briefly, 200 g of powder was macerated in 1L ethanol for 2 days with frequent stirring for the extraction of ginger active ingredients. The extract was then filtered through a filter paper (Whatman No.1). The filtrate was evaporated using a rotary evaporator under reduced pressure at 40 °C. The dried extract was stored at 20 °C for subsequent preparation of the required doses.

### 4.2. Animal Experiment

Animal experiments were carried out at the Department of Medical Parasitology, Faculty of Medicine, Alexandria University, Egypt. *T. gondii* cysts, used for infection, were prepared from the brains of 8-week *T. gondii*-infected mice. The mice brains were isolated, placed in sterile PBS (1 mL PBS/brain), and homogenized in a tissue homogenizer (Wheaton, IL, USA). Homogenates were combined and cysts were then counted using a hemocytometer under 400× magnification. The brain suspension was then diluted to a concentration of 100 cysts/mL which was then used for the infection. Next, 0.1 mL containing 10 cysts was used for the infection of each mouse according to previous studies [71].

In our experiment, we used 40 laboratory-bred male Swiss albino mice (six to seven weeks old). All mice were kept in well-ventilated cages, provided with water and standard pellet food, and maintained under controlled conditions of light (12 h light/12 h dark) and temperature (25 ± 2 °C).

To ensure the absence of any parasitic infections, stools were carefully examined, for three consecutive days, using a direct wet mount smear, iodine-stained smears, a fecal flotation technique [72], and a formol-ether concentration method [73]. Additionally, a modified Ziehl-Neelsen technique was used to confirm the absence of acid-fast bacteria [74].

Briefly, a stool specimen from each mouse was mixed with two drops of saline on a glass slide then directly examined under the microscope or after the addition of one drop of Lugol’s iodine to an air-dried smear. Stool specimens were further examined for parasitic forms using a fecal flotation solution (Sheather’s solution; for most common parasite eggs and oocysts) and a formol-ether sedimentation technique (to detect trematode eggs and protozoan cysts), as previously described. In addition, air-dried smears prepared from stool concentrates were fixed with methanol then stained with Ziehl-Neelsen stain for the detection of any acid-fast bacteria [75].

The mice were divided into four groups (10 mice each). One group was not infected (uninfected), whereas three groups were orally inoculated with 0.1 mL of brain homogenate (10 cysts) for 6 weeks to induce chronic toxoplasmosis [41,71].

Six weeks post-infection, we treated one infected group of mice with spiramycin (100 mg/kg/day) at a fixed hour daily for 10 days; positive control [76,77], and another group with ginger extract (250 mg/kg/day) orally for 2 weeks [43]. One infected group of mice was left untreated (infected untreated, negative control). Both spiramycin and ginger extract were dissolved in water and administrated orally [76]. At the end of the experiment (60 days PI), all mice were anesthetized with isoflurane by the inhalation route and euthanized by cervical dislocation followed by the isolation of organs for the evaluation of the therapeutic efficacy of ginger extract compared to negative and positive controls [76].

### 4.3. Evaluation of Ginger Extract Treatment Efficacy against T. gondii Infection

#### 4.3.1. Quantification of Parasite Burden in Mice Brains

All mice were sacrificed at the end of the experiment (60 days PI) and their brains were removed. Five brains from each mouse group were used for counting cysts and the other five brains were fixed in 10% formaldehyde in PBS and kept for histopathological studies [78]. Each of the five brains/group was rinsed in PBS, weighed, and then homogenized in 1 mL PBS (Omni TH-220) for 5 min. A 0.1 mL of the homogenate was spread on a clean slide, air dried, fixed in methanol, and stained with Giemsa stain (Merck, Darmstadt, Germany) for 30–45 min. Slides were then washed with water, dried, and the total number of cysts was counted and multiplied by 10 to get the number of cysts/mouse brain. The mean number of the cysts/group was then calculated for comparison between mice groups [78].

#### 4.3.2. Histopathological Examination

##### Hematoxylin and Eosin Staining

The brain, liver and lung tissues of mice from different groups were collected, fixed in 10% formalin in PBS, dehydrated in ascending grades of ethanol, embedded in paraffin, cut into 5 µm serial sections, and stained with Hematoxylin and Eosin (H & E) stain [42]. Standard light microscopy was used for histopathological examination by a blinded independent pathologist.

Brain tissue sections were microscopically evaluated for shrunken cells, cells with vacuolated cytoplasm, interstitial edema, congested blood vessels, the area of necrosis, inflammatory infiltrate, and hemorrhage. Lung tissues were examined for the presence of inflammatory infiltrations in the peribronchial, perivascular and alveolar wall, epithelial desquamation and macrophages in the alveolar spaces, parenchymal fibrosis, and emphysematous areas. Additionally, the lung parenchyma was examined for distorted appearance with loss of alveolar architecture, cellular infiltration, and thickened septa. The liver tissue was examined for portal tract inflammation, lytic necrosis, apoptosis and focal inflammation.

##### Immunohistochemistry

Four µm-thick sections were cut from paraffin-embedded brain tissues and placed on saline-coated glass slides, which were then incubated overnight at room temperature. The slides were deparaffinized in xylene for 20 min, then rehydrated in decreasing ethanol concentrations (100% ethanol for 5 min, 2 min in each of 80%, 70%, and 50% ethanol) before being rinsed in distilled water. The tissue sections were incubated in 0.6% H_2_O_2_ for 10 min to inhibit endogenous peroxidase activity. The tissue sections were then rinsed twice with PBS before being boiled twice in Tris/EDTA buffer (pH = 9.0) in a microwave oven at mid–high power for 10 min, to retrieve antigens, then allowed to cool to room temperature for 30 min.

Tissue sections were washed twice with PBS then treated with superblock and incubated overnight at room temperature with Caspase-3 rabbit polyclonal antibody (Catalog no. A11953, ABclonal, Woburn, MA, USA). The excess reagent was removed, and tissue sections were rinsed twice in PBS with 0.05% Tween-20 (PBS-T). The detection kit was the Mouse/Rabbit ImmunoDetector DAB HRP Brown Detection System (ready to use) (BIO SB, Santa Barbara, CA 93117, USA. Catalog no.: BSB 0003). A chromogenic reaction was carried out with DAB substrate.

All slides were then counterstained with hematoxylin for 30 s, washed with running water, dehydrated in graded ascending series of alcohols (70%, 80%, 90%, and 100%), and cleared in xylene for 5 min. Finally, all slides were mounted with DPX, cover-slipped, imaged at 400× magnification and scanned. Images were processed using ImageJ scanner and viewer software (LOCI, University of Wisconsin, USA).

Cerebral cortical neurons with cytoplasmic reaction to antibodies were considered caspase-3 positive cells. The number of caspase-3 positive cells was counted in 3 different high-power fields (HPF) and the mean was calculated. The mean number of caspase 3-positive cells/HPF in each mice group was then calculated for comparison between groups.

### 4.4. Statistical Analysis

Statistical analysis was performed using SPSS-16 and one-way analysis of variance (ANOVA) test. Differences between groups were considered statistically significant at *p* < 0.05.

## 5. Conclusions

The findings showed that treatment with ginger extract reduced the parasite burden in the brains of mice infected with the *T. gondii* ME-49 strain during the chronic phase of infection. Ginger extract treatment reversed *T. gondii*-induced pathological changes in the brain, liver, and lungs. These findings indicate that ginger extract could be a potential alternative therapeutic for treating chronic toxoplasmosis. Further studies are needed to determine the most active ingredient in the extract and to mechanistically understand the anti-toxoplasmosis effects of ginger extract, which could be useful in developing more effective therapeutic derivatives.

## Data Availability

Not applicable.
